# Identification of the Hydrogen Uptake Gene Cluster for Chemolithoautotrophic Growth and Symbiosis Hydrogen Uptake in *Bradyrhizobium Diazoefficiens*

**DOI:** 10.1264/jsme2.ME15182

**Published:** 2016-02-25

**Authors:** Sachiko Masuda, Masaki Saito, Chiaki Sugawara, Manabu Itakura, Shima Eda, Kiwamu Minamisawa

**Affiliations:** 1 Graduate School of Life Sciences, Tohoku UniversityKatahira, Aoba-ku, Sendai, Miyagi 980–8577Japan

**Keywords:** *Bradyrhizobium diazoefficiens*, chemolithoautotrophic growth, hydrogenase, symbiosis

## Abstract

The hydrogen uptake (Hup) system of *Bradyrhizobium diazoefficiens* recycles the H_2_ released by nitrogenase in soybean nodule symbiosis, and is responsible for H_2_-dependent chemolithoautotrophic growth. The strain USDA110 has two *hup* gene clusters located outside (locus I) and inside (locus II) a symbiosis island. Bacterial growth under H_2_-dependent chemolithoautotrophic conditions was markedly weaker and H_2_ production by soybean nodules was markedly stronger for the mutant of *hup* locus I (*ΔhupS_1_L_1_*) than for the mutant of *hup* locus II (*ΔhupS_2_L_2_*). These results indicate that locus I is primarily responsible for Hup activity.

Soybean bradyrhizobia have two lifestyles: as symbiotic bacteroids that fix atmospheric nitrogen in host plants or as free-living soil bacteria. As a symbiont, *B. diazoefficiens* synthesizes a hydrogen uptake (Hup) system that recycles the H_2_ formed as a byproduct of nitrogenase activity (4). This symbiotic hydrogen oxidation increases nitrogen fixation efficiency, thereby enhancing the productivity of the legume host (3, 6). As free-living cells, *Bradyrhizobium diazoefficiens* Hup^+^ strains have the ability to grow chemolithoautotrophically by using H_2_ as an electron donor (7).

Two sets of *hup* genes have been identified in *B. diazoefficiens*: a large cluster outside the symbiosis island (*hup* locus I, genome position 7,620,025–7,645,755) and a small cluster inside the symbiosis island (*hup* locus II, genome position 1,888,916–1,902,575) (6, 11). A transcriptome analysis previously showed that several *hup* genes located outside the symbiosis island were up-regulated during H_2_-dependent chemolithoautotrophic growth, whereas several *hup* genes located inside the symbiosis island were up-regulated in symbiotic bacteroids (1, 6). These findings imply that *hup* locus I plays an important role in chemolithoautotrophic growth, while symbiotic *hup* locus II may contribute to Hup activity in the nodules. In the present study, we constructed *hupSL* deletion mutants in order to clarify the contribution of each *hup* gene cluster during the chemolithoautotrophic growth and nodulation of *B. diazoefficiens* USDA110.

## Materials and Methods

The strains and plasmids used in this study are listed in Table 1. The HM salt medium for the preculture and Hup medium for chemolithoautotrophic growth were described previously (14). Antibiotics were added to the media for *B. diazoefficiens* USDA110 and *Escherichia coli* strains as described previously (13).

We generated *hupS_1_L_1_* and *hupS_2_L_2_* deletion mutants as follows. A DNA fragment containing *hupFDCL_1_S_1_V* was isolated from brp15657, a plasmid from the pUC18 clone library of *B. diazoefficiens* USDA110 sequences (11), and inserted into the *Hin*dIII site of pK18mob. The resultant plasmid, pK18mob-*hup*I, was digested with *Apa*I and ligated with the *Apa*I/*Eco*RI adaptor, yielding pK18mob-*hup*Iad. The omega cassette isolated from pHP45Ω was inserted at the *Eco*RI site of pK18mob-*hup*Iad, yielding pK18mob-*hup*Iomega. Triparental mating using pRK2013 was performed as described previously (13). A similar strategy was used to construct the *hupS_2_L_2_* deletion mutant. Briefly, *hupKHFDCL_2_S_2_* was isolated from brp07423, and inserted into pK18mob, yielding pK18mob-*hup*II. pK18mob-*hup*II was ligated with the *Eco*O109I/*Bam*HI adaptor, resulting in pK18mob-*hup*IIad. The Tc^r^ cassette was isolated from p34S-Tc and inserted into the *Bam*HI site of pK18mob-*hup*IIad, yielding pK18mob-*hup*IITc. The double crossover events of these deletion mutations were verified by a Southern blot analysis.

The inoculants were prepared as described previously (14). Aliquots (10 μL) of the cells (OD_660_ at 0.1) were streaked on Hup medium, and the agar plates were statically incubated at 25°C for 14 d in an atmosphere containing 1% O_2_, 5% CO_2_, 10% H_2_, and 84% N_2_.

Soybean seedlings (*Glycine max* cv. Enrei) grown in a plant box in a growth chamber were inoculated with *B. diazoefficiens* as described previously (8, 9). The nodulated roots were transferred into a 300-mL bottle 30 d later, and a 0.5-mL gas sample from the head space of the bottle was injected into a GC-2014 gas chromatograph (Shimadzu, Kyoto, Japan) as described previously (13). The flow rate of the carrier gas (N_2_) was 30 mL min^−1^.

## Results

**Fig. 2:**
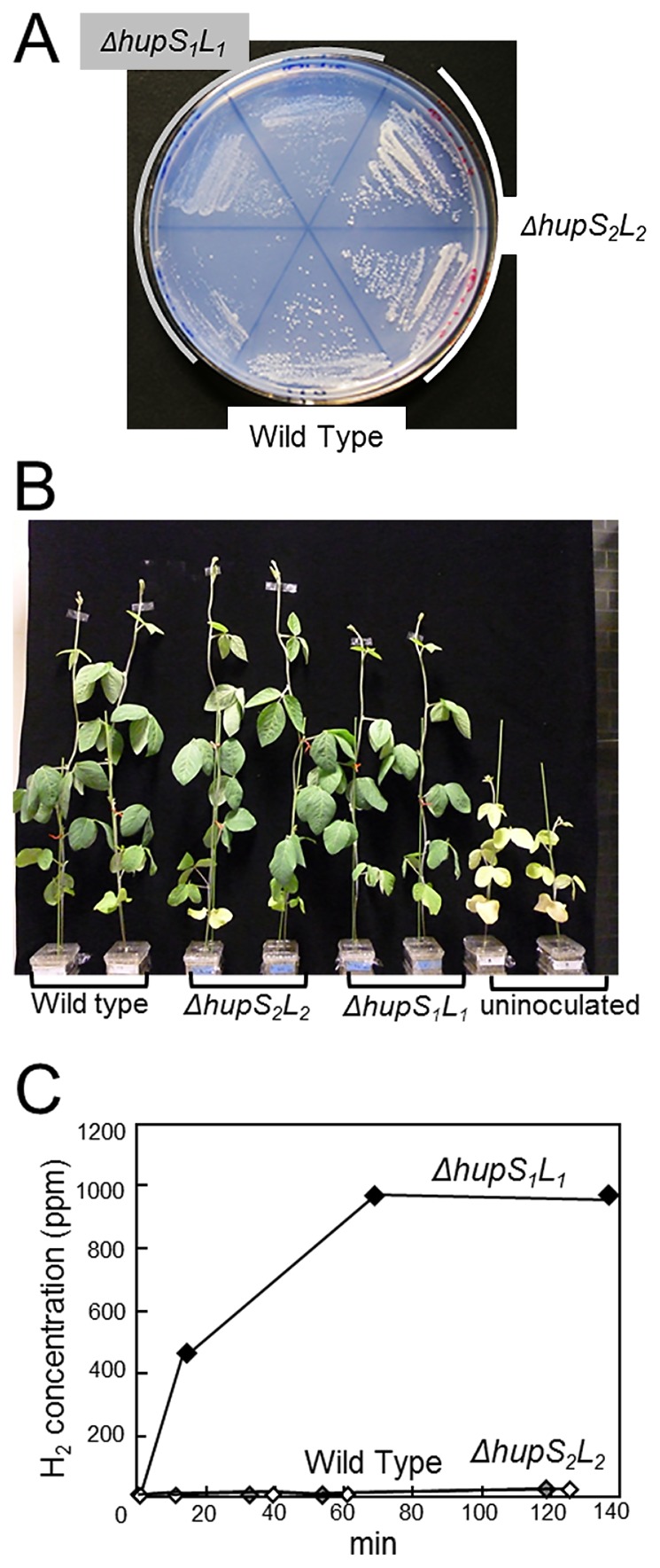
Comparison of chemolithoautotrophic growth (A) and symbiotic phenotypes (BC) between *hup* mutants and the wild-type strain of *B. diazoefficiens* USDA110. (A) Growth phenotype on Hup agar medium under an atmosphere of 84% N_2_, 10% H_2_, 5% CO_2_, and 1% O_2_ at 25°C. (B) Plant growth 30 d after inoculation. (C) Concentration of H_2_ produced by the root nodules. White squares, wild type; black squares, *ΔhupS_1_L_1_* mutant; grey squares, *ΔhupS_2_L_2_* mutant.

Wild-type USDA110 and the *ΔhupS_2_L_2_* mutant grew on Hup agar medium under chemolithoautotrophic growth conditions (Fig. 2A). However, the *ΔhupS_1_L_1_* mutant showed markedly weaker growth than that of the wild type on this medium (Fig. 2A). The height of plants inoculated with *ΔhupS_1_L_1_* appeared to be lower than those inoculated with the wild type and *ΔhupS_2_L_2_* mutant (Fig. 2B); however, no significant differences were observed in plant dry weights or fresh nodule weights (Table 2). H_2_ was not produced from the nodulating roots of the wild type or *ΔhupS_2_L_2_* mutant (Fig. 2C), indicating that Hup activity compensated for the production of H_2_ via nitrogenase. In contrast, H_2_ was produced by the *ΔhupS_1_L_1_* nodules (6.4 μmol h^−1^ g fresh nodule weight^−1^) (Fig. 2C), indicating that Hup activity was lower than the production of H_2_ via nitrogenase. These results suggest that *hup* genes outside the symbiosis island are the primary cluster involved in chemolithoautotrophic growth and Hup activity in the nodules.

## Discussion

In the present study, the mutation of *hupS_2_L_2_* did not change nodule H_2_ production from that by wild-type USDA110 (Fig. 2C), even though some genes on *hup* locus II were up-regulated in symbiotic bacteroids (1, 6). *Hup* locus I contains a complete set of the *hup*-*hyp*-*hox* cluster, and is missing from *hup* locus II (11). Thus, *hupS_2_L*_2_ in locus II may not be fully induced without the *hup* gene assemblage, resulting in no or weak Hup activity by the *hupS_2_L*_2_ genes. On the other hand, the *hup* gene cluster outside the symbiosis island, which we identified as the primary *hup* gene cluster contributing to Hup activity in free-living and symbiotic cells, is located on a typical genomic island (trnM element) of *B. diazoefficiens* USDA110. The trnM element is likely acquired in the USDA110 lineage after the divergence of strains USDA110 and USDA6^T^ because *B. japonicum* USDA6^T^ completely lacks this element (10, 11, 12).

*Hup* genes were also found in the symbiosis island of the USDA6^T^ genome even though USDA6^T^ was previously reported to exhibit no Hup activity (10, 11, 12). The *hup* genes in USDA6^T^ on the symbiosis island had 99–100% amino acid sequence identity to the corresponding genes in *hup* locus II of USDA110. In contrast, USDA6^T^ *hup* genes had only 43–83% amino acid sequence identity to genes in *hup* locus I of USDA110, which is similar to the homology (45–83%) between *hup* genes in loci I and II of USDA110. These results suggest that *hup* genes on locus II of USDA110 and *hup* genes in USDA6^T^ do not contribute to the Hup activities of these strains and appear to be derived from the acquisition of symbiosis islands. Therefore, our results imply the horizontal gene transfer of the primary *hup* cluster via the genomic island to the lineage of *B. diazoefficiens* rather than symbiosis island transfer.

## Figures and Tables

**Fig. 1:**
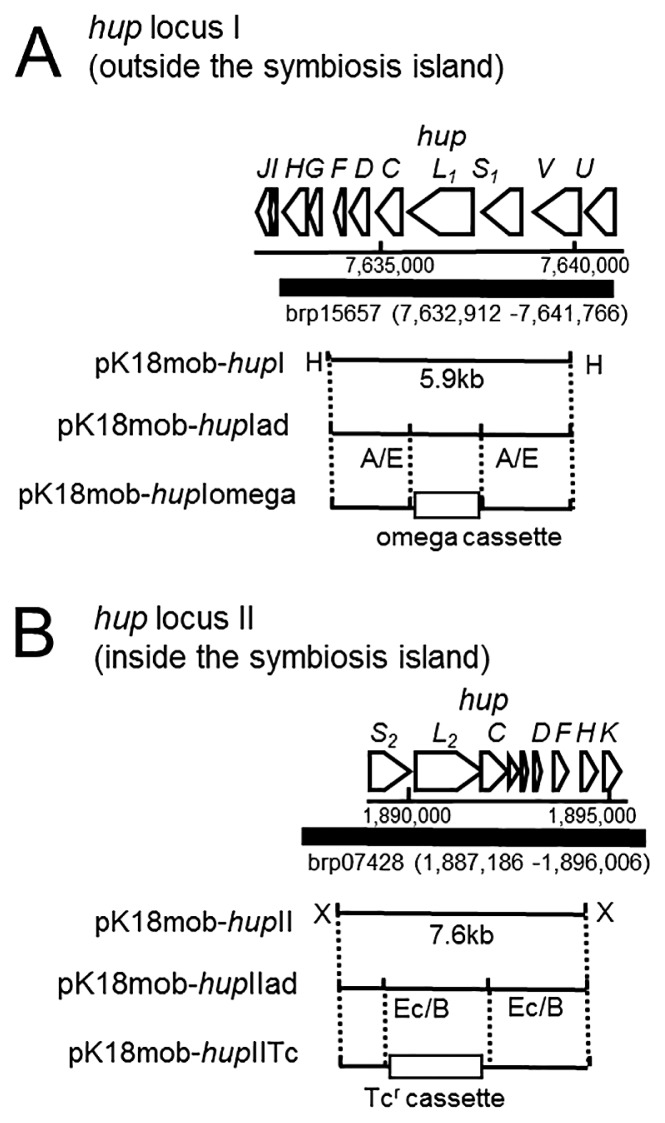
Gene maps of *hup* gene clusters in *B. diazoefficiens*. (A) Gene map of *hup* locus I showing the genome position of *hup* locus I and brp15657. pK18mob-*hup*I carries a 5.9-kb DNA fragment containing *hupFDCL_1_S_1_V* genes. pK18mob-*hup*Iad was ligated with an *Apa*I/*Ec*oRI adaptor. pK18mob-*hup*Iomega had an omega cassette, which was inserted at the *Eco*RI site of pK18mob-*hup*Iad. (B) Gene map of *hup* locus II showing the genome position of *hup* locus II and brp07428. The strategy used to construct the *hupS_2_L_2_* deletion mutant is shown as the *hupS_1_L_1_* deletion mutant. H, *Hin*dIII; A, *Apa*I; E, *Eco*RI; X, *Xho*I; Ec, *Eco*O109I; B, *Bam*HI, Tc^r^, tetracycline-resistant.

**Table 1:** Strains and plasmids used in the present study.

Strain or plasmid	Relevant characteristics^a^	Reference or source
*Bradyrhizobium diazoefficiens*		
USDA110	Soybean bradyrhizobia, wild type	11
*ΔhupS_1_L_1_*	USDA110 *hupS_1_L_1_*::*aadA*; Sm^r^ Sp^r^	This study
*ΔhupS_2_L_2_*	USDA110 *hupS_2_L_2_*::*tet*; Tc^r^	This study
*Escherichia coli*		
DH5α	cloning strain	Toyobo Inc.^a^
Plasmids		
p34S-Tc	Plasmid carrying a 2.1-kb Tc cassette; Tc^r^	2
pHP45Ω	Plasmid carrying a 2.1-kb Ω cassette; Sp^r^ Sm^r^ Ap^r^	15
pK18mob-*hup*I	pK18mob carrying a 5.9-kb *hupFDCL_1_S_1_VU Hin*dIII fragment of brp15657; Km^r^	This study
pK18mob-*hup*Iad	pK18mob-*hup*I with an *Apa*I/*Eco*RI adaptor; Km^r^	This study
pK18mob-*hup*Iomega	pK18mob carrying *hupS_1_L_1_*::*aadA*; Sm^r^ Sp^r^ Km^r^	This study
pK18mob-*hup*II	pK18mob carrying a 7.6-kb *hupS_2_L_2_CDFHK Xho*I fragment of brp07428; Tc^r^	This study
pK18mob-*hup*IIad	pK18mob-*hup*II with an *Eco*O109I/*Bam*HI adaptor; Km^r^	This study
pK18mob-*hup*IITc	pK18mob carrying *hupS_2_L_2_*::*tet*; Tc^r^ Km^r^	This study
pK18mob	cloning vector; pMB1*ori* oriT; Km^r^	16
pRK2013	ColE1 replicon carrying RK2 transfer genes; Km^r^	5
brp15657	pUC18 carrying *hupUVS_1_L_1_CDFG*	11
brp07423	pUC18 carrying *hupKHFDCL_2_S_2_*	11

Ap^r^, ampicillin-resistant; Tc^r^, tetracycline-resistant; Km^r^, kanamycin-resistant; Sm^r^, streptomycin-resistant; Sp^r^, spectinomycin-resistant.

a Osaka, Japan.

**Table 2:** Plant dry weights and fresh nodule weights of inoculated wild-type USDA110 and mutants.

Strains	Plant dry weight (g)	Fresh nodule weight (g)
USDA110	6.9 ± 0.9*^a^*	1.36 ± 0.20*^a^*
*ΔhupS_1_L_1_*	6.8 ± 1.1*^a^*	1.38 ± 0.20*^a^*
*ΔhupS_2_L_2_*	6.9 ± 0.6*^a^*	1.29 ± 0.13*^a^*

Plants were harvested 30 d after inoculation. Values are presented as an average and standard deviation (*n*=5). Tukey’s multiple comparison test was used for statistical analyses (*p*<0.05).
